# Biostatistics for Evidence‐Based Medicine: A Practical Review for Clinicians

**DOI:** 10.1111/jep.70682

**Published:** 2026-10-18

**Authors:** Mayura Thilanka Iddagoda

**Affiliations:** ^1^ Royal Perth Hospital Perth Western Australia Australia; ^2^ The University of Western Australia Perth Western Australia Australia

**Keywords:** biostatistics, clinical research, evidence‐based medicine, hypothesis, research methodology, statistical analysis

## Abstract

**Background:**

Biostatistics forms the foundation of evidence‐based medicine by providing the methods required to design, analyse, interpret, and critically appraise clinical research. Despite its importance, many clinicians find statistical concepts difficult to understand and apply in everyday practice.

**Aim:**

This review provides a practical overview of the fundamental principles of biostatistics relevant to clinicians involved in research or the interpretation of medical literature.

**Design:**

and content: Key concepts discussed include the hierarchy of evidence, development of research questions and hypotheses, population and sample selection, sample size determination and statistical power, variable classification, and the distinction between descriptive and inferential statistics. The article also introduces the principles of data organisation, univariate, bivariate, and multivariable analyses, and the factors influencing the selection of appropriate statistical models based on study design, variable type, and data characteristics. Throughout, the emphasis is on understanding the rationale behind statistical methods rather than on mathematical derivations, enabling clinicians to critically evaluate published studies and communicate effectively with statisticians.

**Conclusion:**

By providing a structured introduction to core biostatistical concepts, this review aims to improve statistical literacy, facilitate evidence‐based clinical decision‐making, and support clinicians in conducting and interpreting high‐quality clinical research.

## 1 Introduction

Modern medicine is founded on the principles of evidence‐based medicine (EBM), in which clinical decisions are guided by the best available scientific evidence rather than by individual opinion or tradition [1]. Evidence is generated through well‐designed clinical research, including observational studies, randomised controlled trials, and systematic reviews [1].

However, not all research provides evidence of equal quality. Clinicians must be able to critically appraise the validity, reliability, and applicability of published studies before incorporating their findings into clinical practice. Biostatistics provides the tools required to design studies, analyse data, quantify uncertainty, and determine whether observed findings are likely to represent true effects rather than chance [2]. A sound understanding of basic statistical principles enables clinicians to interpret medical literature appropriately, assess the strength of evidence, and make informed decisions for patient care. The aim of this article is to provide an insight into the basics of biostatistics for clinical research. The paper will cover structured topics: level of evidence, hypothesis development, study design, and basic analytical models.

## 2 Level of Evidence

The strength of medical evidence is commonly described using a hierarchy of evidence, which ranks study designs according to their ability to minimise bias and provide reliable conclusions. (See Table 1) At the top of this hierarchy are systematic reviews [3] and meta‐analyses of high‐quality randomised controlled trials (RCTs) [4]. These studies systematically identify, critically appraise, and statistically combine results from multiple studies, providing the most comprehensive and precise estimate of an intervention's effect. The next highest level of evidence is provided by randomised controlled trials, which are considered the gold standard for evaluating treatments because random allocation reduces selection bias and confounding. RCTs employing double blinding, in which neither participants nor investigators know which treatment is being administered, further minimise performance and assessment bias, yielding more reliable results. Below are RCTs and observational studies, including prospective and retrospective cohort studies and case‐control studies. These designs are valuable when randomisation is impractical or unethical but are more susceptible to confounding and bias, limiting their ability to establish causal relationships. Cross‐sectional studies provide useful information on disease prevalence and associations at a single point in time but cannot determine temporal or causal relationships. Case series and case reports describe individual patients or small groups and are particularly useful for identifying new diseases, rare conditions, or unexpected adverse events, although they provide limited evidence of treatment effectiveness. At the base of the hierarchy are expert opinion, narrative reviews, and laboratory or animal studies, which are valuable for generating hypotheses and guiding future research but provide the lowest level of evidence for clinical decision‐making. Understanding this hierarchy enables clinicians to critically appraise medical literature and appropriately weigh the strength of evidence when making evidence‐based clinical decisions [4].

**Table 1:** Level of evidence based on study designs.

Level of evidence	Study design	Strengths
Level I	Systematic reviews and meta‐analyses of randomised controlled trials (RCTs)	Highest level of evidence, which combines results from multiple high‐quality studies, increasing statistical power and precision.
Level II	Randomised controlled trials (RCTs)	Gold standard for evaluating interventions. Randomisation reduces selection bias, while blinding minimises performance and assessment bias.
Level III	Non‐randomised controlled studies (cohort studies and case‐control studies)	Useful when RCTs are impractical or unethical. Can identify associations but are more susceptible to confounding and bias.
Level IV	Case series and case reports	Describe clinical experiences and may identify new diseases or adverse events but cannot establish causation.
Level V	Expert opinion, narrative reviews, and laboratory research	Lowest level of clinical evidence, and this is valuable for generating hypotheses but provides limited evidence for clinical decision‐making.

## 3 Synthesis of a Hypothesis

A research question is the starting point of a research study. It identifies the problem or issue to be investigated and defines what the researcher wants to answer. A good research question is clear, focused, relevant, and feasible, guiding the selection of the study design, participants, variables, and methods of analysis. Research questions are often broad and exploratory [5].

A hypothesis, on the other hand, is a specific, testable prediction derived from the research question. A hypothesis is a clear, testable statement or prediction about a relationship, difference, or association within a population [5, 6]. Because it is usually impractical to study an entire population as described previously, researchers test the hypothesis using a sample that is representative of that population. The data collected from the sample are analysed using inferential statistical methods to determine whether there is sufficient evidence to support or reject the hypothesis. The results obtained from the sample are then used to make inferences about the wider population [6]. (See Figure 1 and next section on population and sample).

In hypothesis testing, two competing hypotheses are defined. The common hypothesis is called the null hypothesis (H_0_). The H_0_ assumes there is no difference, no association, or no treatment effect in the population, and any observed difference in the sample is assumed to be due to chance alone. On the other hand, the alternative hypothesis (H_1_ or Ha) assumes that a true difference, association, or treatment effect exists in the population. The purpose of statistical testing is not to prove a hypothesis but to determine whether the sample provides sufficient evidence to reject the null hypothesis in favour of the alternative hypothesis [5, 6, 7].

As an example, imagine researchers wish to determine whether a new antihypertensive drug lowers systolic blood pressure more than standard treatment. The population is all patients with hypertension. The sample comprised 200 patients recruited into a clinical trial, and the null hypothesis (H_0_) is that there is no difference in mean systolic blood pressure between the two treatments. The alternative hypothesis (H_1_) will be that the new drug results in a different (or lower) mean systolic blood pressure than standard treatment [7].

## 4 Developing a Study

Once a research hypothesis has been formulated, an appropriate study design is selected to test it. The choice of study design depends on the research question, the feasibility of conducting the study, available resources, ethical considerations, and the type of outcome being investigated. A well‐designed study aims to minimise bias, control for confounding factors, and produce reliable and reproducible results.

### 4.1 Population and Sample

The first step of a study design is defining the sample. In an ideal world, researchers would study the entire population of interest to obtain the true value of a clinical outcome or treatment effect [8]. However, studying every individual in a population is usually impractical due to the large number of people involved, the high cost, time constraints, and logistical challenges. In some situations, such as rare diseases or large national populations, it is simply impossible to collect data from everyone. Therefore, researchers study a sample, a subset of the target population that is representative of the whole population [8, 9]. (See Table 2).

**Table 2:** Differences between population vs sample.

Description	Population	Sample
Definition	Entire group of interest	A subset of the population
Size	Usually very large	Smaller and manageable
Description	Described using parameters	Described using statistics
Limitations	Often impossible to study completely	Used to make conclusions about the population
Example	All adults with diabetes in Australia.	200 adults with diabetes recruited from a hospital clinic
Metrics and standard symbols	Mean (μ), Standard deviation (σ), Variance (σ^2^), Size (N), Proportion (P), Correlation Coefficient (ρ)	Mean (x̄), Standard deviation (s), Variance (s^2^), Size (n), Proportion (p̂), Correlation Coefficient (r)

A well‐designed sample allows researchers to estimate population characteristics accurately while using far fewer resources. The validity of these estimates depends on selecting a sample representative of the population, typically through appropriate sampling methods such as random sampling. Statistical methods are then used to quantify the uncertainty associated with these estimates and to determine how likely the findings are to reflect the true population rather than random variation [8]. Consequently, most clinical research relies on samples to make valid inferences about the broader population to which the results will be applied. (See Figure 1).

**Figure 1:**
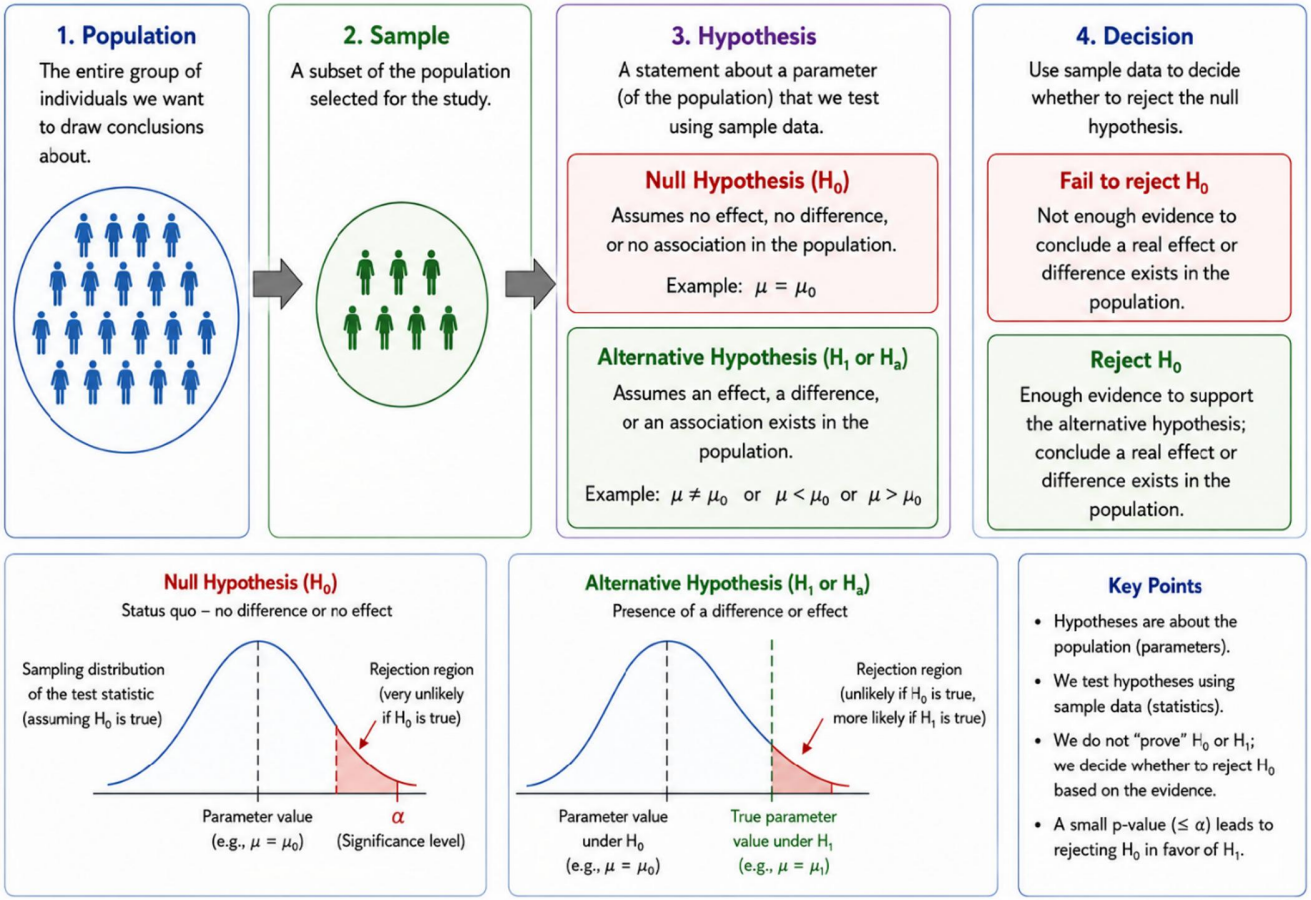
Relationship between the population, sample, and hypothesis testing. The figure illustrates how a representative sample is selected from a target population and used to test a statistical hypothesis. The null hypothesis (H₀) assumes no true difference, effect, or association in the population, whereas the alternative hypothesis (H₁ or Hₐ) assumes that a true difference, effect, or association exists. Statistical analysis of the sample provides evidence to reject or fail to reject the null hypothesis and allows inferences about the wider population.

### 4.2 Sample Size and Power

Determining an appropriate sample size is a critical step in study design. A sample that is too small may fail to detect a true difference (an underpowered study), while an unnecessarily large sample wastes time and resources and may expose more participants to research than necessary [10]. Power calculations are performed before a study begins to estimate the minimum number of participants needed to detect a clinically meaningful effect. Sample size is influenced by several factors, including the expected effect size, the significance level (α), the desired statistical power (1−β), and the variability of the outcome. Most clinical studies are designed with a significance level of 0.05 and a statistical power of 80% or 90%. Performing an appropriate power calculation ensures that a study is scientifically robust, ethically justified, and capable of producing reliable and clinically meaningful results. Nowadays, there are online platforms for power calculations. For basic studies, OpenEpi and ClinCalc are the easiest online tools because they require minimal statistical knowledge. For researchers performing more advanced analyses, G*Power remains the standard software used in many universities and research institutions [9, 10].

## 5 Organising Data in a Sample

A variable is any characteristic or measurement that can take different values among individuals in a study. The type of variable determines how data are collected, summarised, analysed, and presented. Variables are broadly classified as categorical (qualitative) or numerical (quantitative), as shown in Table 3 [11].

**Table 3:** Qualitative versus quantitative variables.

Characteristic	Qualitative (Categorical) variables	Quantitative (Numerical) variables
Definition	Describe characteristics or categories	Represent measurable numerical values
Nature of data	Non‐numeric categories or labels	Numeric values obtained by counting or measurement
Subtypes	Nominal and Ordinal	Discrete and Continuous
Can be ranked?	Ordinal: Yes; Nominal: No	Yes (numerical order)
Arithmetic operations	Not meaningful	Meaningful (addition, subtraction, averages, etc.)
Common summary measures	Frequencies (n) Percentages (%)	Mean ± SD Median and interquartiles Minimum and maximum or range
Common graphical displays	Bar chart, Pie chart	Histogram, Box plot, Scatter plot, Line graph
Examples	Sex, blood group, smoking status, disease stage, frailty category	Age, weight, blood pressure, serum creatinine, length of stay, number of falls
Typical statistical tests	Chi‐square test, Fisher's exact test	*t*‐test, ANOVA, Mann–Whitney *U* test, correlation, regression

Categorical variables describe qualities or groups rather than numerical values. They are divided into nominal and ordinal variables. Nominal variables have categories with no natural order, such as sex (male/female), blood group (A, B, AB, O), or smoking status (smoker/non‐smoker). Ordinal variables have categories with a meaningful order or ranking, but the intervals between categories are not necessarily equal. Examples include pain severity (mild, moderate, severe), frailty status (fit, vulnerable, frail), and cancer stage (I–IV) [11, 12].

Numerical variables represent measurable quantities and can be analysed using arithmetic operations. They are classified as discrete or continuous variables. Discrete variables take whole‐number values and usually represent counts, such as the number of hospital admissions, falls, or medications. Continuous variables can take any value within a range and are measured [13]. Examples include age, body weight, blood pressure, serum creatinine concentration, and hospital length of stay.

Understanding the type of variable is essential because it determines the appropriate methods for data presentation and statistical analysis in the next step. Categorical variables are commonly summarised using frequencies and percentages, whereas numerical variables are summarised using means and standard deviations for normally distributed data or medians and interquartile ranges for skewed data. Similarly, the choice of statistical tests depends largely on the type of variables being analysed [11, 12, 13].

## 6 Analysis

Data analysis begins with data cleaning, which involves identifying missing values, errors, outliers, and inconsistencies to ensure the dataset is accurate and complete [11]. The characteristics of the study population are then summarised using descriptive statistics, such as means, medians, standard deviations, frequencies, and percentages, as described in the previous section and Table 4.

**Table 4:** Descriptive versus inferential statistics.

Descriptive statistics	Inferential statistics
Summarises observed data	Draws conclusions about the population
Describes what was observed	Predicts or generalises beyond the sample
No hypothesis testing	Includes hypothesis testing
No *p*‐values or confidence intervals	Uses *p*‐values and confidence intervals
Examples: mean, median, SD, percentages	Examples: *t*‐test, chi‐square, regression, etc

The next step is inferential statistical analysis, in which appropriate statistical tests are selected based on the study design, variable types, and data distribution. These analyses are used to compare groups, assess associations, evaluate treatment effects, or identify predictors of outcomes. Results are commonly reported using effect estimates, 95% confidence intervals, and *p*‐values to quantify the strength and statistical significance of the findings [11, 14].

### 6.1 Descriptive versus Inferential Statistics

The two main types of statistics used to report sample data are descriptive statistics (What does the sample data show?) and inferential statistics (What can we conclude about the population from this sample?). Together, these two branches form the foundation of statistical analysis in evidence‐based clinical research, as shown in Table 4 [14].

Descriptive statistics are used to organise, summarise, and present data in a meaningful way. They describe the characteristics of the study sample without drawing conclusions beyond the observed data. Common descriptive statistics include measures of central tendency (mean, median, and mode), dispersion (standard deviation, variance, range, and interquartile range), and frequency distributions (counts and percentages). Data are often presented using tables, bar charts, histograms, box plots, or pie charts [14, 15].

In contrast, inferential statistics use sample data to draw conclusions about the wider population. Because only a sample is studied, inferential statistics account for sampling variability and quantify the uncertainty of the results. They are used to test hypotheses, compare groups, estimate treatment effects, and identify associations or predictors. Common inferential statistical methods include *t*‐tests, chi‐square tests, analysis of variance (ANOVA), correlation, regression analysis, confidence intervals, and *p*‐values [11, 14, 15, 16].

### 6.2 Selecting Statistical Analysis Model

Selecting an appropriate statistical model is essential for obtaining valid and reliable results. The choice depends on multiple factors and is complicated. It is usually advisable to get an experienced statistician's opinion. The common predictors are study aim and design, variable types, and the characteristics and distribution of the data. Before analysis, researchers should assess whether the data are categorical or numerical, whether numerical data are normally distributed or skewed, and whether observations are independent or paired. These characteristics determine whether parametric or non‐parametric statistical methods are appropriate [11, 12, 13, 14, 15, 16].

It is important to know the number of variables in the analysis. (See Table 5) Univariate analysis describes a single variable [12]. It is used to describe the characteristics of a single variable, such as its frequency, percentage, mean, or median. Whereas bivariate analysis investigates the relationship or difference between two variables. Bivariate analysis is used to determine whether an association or difference exists between variables and forms the basis of many inferential statistical tests. The choice of analysis depends on the type of variables being compared. For example, a chi‐square test is used to examine the association between two categorical variables; a *t*‐test or ANOVA is used to compare the mean of a continuous variable between groups; and a correlation assesses the relationship between two continuous variables. Bivariate analysis is often the first step in testing research hypotheses before progressing to multivariable statistical models [14, 15, 16].

**Table 5:** Analysis based on number of variables of interest.

Type of analysis	Number of variables	Example
Univariate	One	What is the mean age of the study participants?
Bivariate	Two	Is age associated with hospital length of stay?
Multivariable	Three or more	After adjusting for age, sex, frailty, and injury severity, which factors independently predict mortality?

Choosing the correct statistical model ensures that the assumptions of the analysis are met and that study conclusions are accurate and clinically meaningful. Figure 2 is an attempt to give an insight into selecting the correct statistical model. The first step in statistical analysis is to clearly define the research question, including the outcome of interest, predictor variables, study design, and potential confounders. The outcome variable is then classified by type (categorical, numerical, time‐to‐event, count, or repeated measures), followed by an assessment of key data characteristics, such as the number of comparison groups and the data distribution. These factors guide the selection of the most appropriate statistical test or regression model, ensuring that the analysis is valid and the study conclusions are reliable [16].

**Figure 2:**
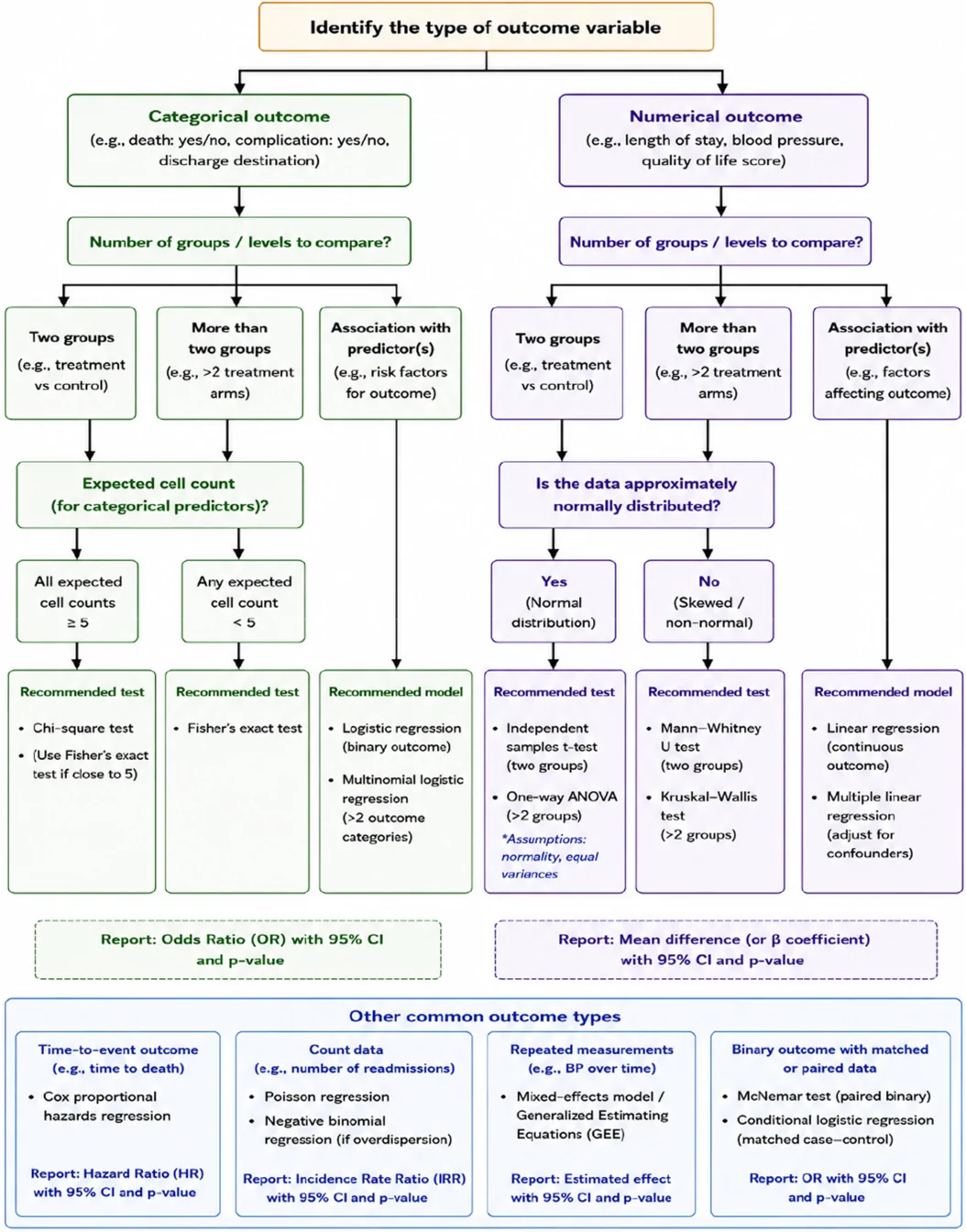
Selection of an appropriate statistical model. This figure provides a simplified approach to selecting a statistical method based on the type of outcome variable and the characteristics of the data. Categorical outcomes may be analysed using chi‐square or Fisher's exact tests for group comparisons and logistic regression for modelling associations. For numerical outcomes, the choice between parametric and non‐parametric methods depends on the number of groups and distribution of the data. The figure also outlines commonly used approaches for time‐to‐event outcomes, count data, repeated measurements, and matched or paired data. BP, Blood pressure; ANOVA, Analysis of variance.

For example, normally distributed continuous data are often analysed using parametric tests, such as the Student's *t*‐test or analysis of variance (ANOVA), whereas skewed continuous data may require non‐parametric tests, such as the Mann–Whitney *U* test or Kruskal–Wallis test [17]. Categorical variables are typically analysed using the chi‐square test or Fisher's exact test. More advanced statistical models, including linear regression, logistic regression, and survival analysis, are selected according to the type of outcome being studied and the need to adjust for potential confounding factors [17, 18, 19].

## 7 Conclusion

Biostatistics is a fundamental component of evidence‐based medicine and underpins every stage of clinical research, from formulating a research question and designing a study to analysing data and interpreting results. Although statistical methods can appear complex, understanding core concepts such as levels of evidence, hypothesis development, sampling, variable types, descriptive and inferential statistics, and the principles guiding statistical model selection enables clinicians to critically appraise medical literature and apply research findings appropriately in clinical practice. This overview provides a practical foundation for clinicians beginning to engage with research and should facilitate more informed interpretation of published studies and collaboration with biostatisticians in future research.

## Conflicts of Interest

The author declares no conflicts of interests.

## Data Availability

Data sharing not applicable to this article as no datasets were generated or analysed during the current study.
